# DDB2 modulates TGF-β signal transduction in human ovarian cancer cells by downregulating NEDD4L

**DOI:** 10.1093/nar/gkv667

**Published:** 2015-06-29

**Authors:** Ran Zhao, Tiantian Cui, Chunhua Han, Xiaoli Zhang, Jinshan He, Amit Kumar Srivastava, Jianhua Yu, Altaf A. Wani, Qi-En Wang

**Affiliations:** 1 Division of Radiobiology, Department of Radiology, The Ohio State University Medical Center, Columbus, OH 43210, USA; 2 Center for Biostatistics, The Ohio State University Wexner Medical Center, Columbus, OH 43210, USA; 3 Comprehensive Cancer Center, The Ohio State University Wexner Medical Center, Columbus, OH 43210, USA

## Abstract

The expression of DNA damage-binding protein 2 (DDB2) has been linked to the prognosis of ovarian cancer and its underlying transcription regulatory function was proposed to contribute to the favorable treatment outcome. By applying gene microarray analysis, we discovered neural precursor cell expressed, developmentally downregulated 4-Like (NEDD4L) as a previously unidentified downstream gene regulated by DDB2. Mechanistic investigation demonstrated that DDB2 can bind to the promoter region of *NEDD4L and* recruit enhancer of zeste homolog 2 histone methyltransferase to repress *NEDD4L* transcription by enhancing histone H3 lysine 27 trimethylation (H3K27me3) at the NEDD4L promoter. Given that NEDD4L plays an important role in constraining transforming growth factor β signaling by targeting activated Smad2/Smad3 for degradation, we investigated the role of DDB2 in the regulation of TGF-β signaling in ovarian cancer cells. Our data indicate that DDB2 enhances TGF-β signal transduction and increases the responsiveness of ovarian cancer cells to TGF-β-induced growth inhibition. The study has uncovered an unappreciated regulatory mode that hinges on the interaction between DDB2 and NEDD4L in human ovarian cancer cells. The novel mechanism proposes the DDB2-mediated fine-tuning of TGF-β signaling and its downstream effects that impinge upon tumor growth in ovarian cancers.

## INTRODUCTION

Ovarian cancer is the most lethal malignancy of the female reproductive tract with a low 5-year survival rate of only 27% in distant stages (1). The American Cancer Society estimates that in 2015, about 21 290 new cases of ovarian cancer will be diagnosed and 14 180 women will die of ovarian cancer in the United States (1). Advanced stage at diagnosis and high tumor relapse result in poor prognosis for most ovarian cancer patients and leading to the highest mortality rate among all gynecological malignancies. Limited by an incomplete understanding of the molecular pathways governing ovarian cancer progression, it remains a major challenge to improve the survival outcome in the clinical practice and hence entails further efforts in identifying key molecular drivers of ovarian cancer progression.

DNA damage-binding protein 2 (DDB2) has been considered a tumor suppressor based on the findings that DDB2-/- mice were not only susceptible to UV-induced carcinogenesis, but also developed spontaneous malignant tumors at a high rate (2,3). The analysis of publicly available datasets indicates that low *DDB2* mRNA expression correlates with poor outcome of ovarian cancer patients (4). Indeed, this kind of correlation can also be found in breast (5) and lung cancer patients (http://www.kmplot.com). In addition, DDB2 has been shown to suppress the tumorigenicity of both ovarian cancer cells (4) and colorectal cancer cells (6). DDB2 is also able to inhibit metastasis of colon cancer (6) and limit the invasiveness of breast cancer (5). Therefore, it is believed that DDB2 plays an important role in impeding tumor progression and tumor relapse. Beyond its well-established function in global genome nucleotide excision repair (7), DDB2 is recognized as a transcriptional regulator for a spectrum of important genes including superoxide dismutase (MnSOD, *SOD1*), IĸB (*NFKBIA*), Bcl-2 (*BCL2*) as well as the key activators of Epithelial–Mesenchymal Transition (EMT) including VEGF, Zeb1 and Snail (5,6,8–10). This transcriptional regulatory function of DDB2 has been suggested to be partially responsible for its tumor suppressing potential.

Given the function of DDB2 in inhibiting ovarian cancer cell survival and sensitizing ovarian cancer cells to cisplatin treatment (9,11), we sought to identify new target genes of DDB2 which function in key signal transduction pathways. Our transcriptome analysis led to the identification of neural precursor cell expressed, developmentally downregulated 4-like (*NEDD4L*) as a new target gene of DDB2. NEDD4L was previously known to be closely related to hypertension control, as it is responsible for triggering ubiquitin-mediated lysosome endocytosis of kidney epithelial sodium channels (ENaC) subunits (12,13). It has also been reported that NEDD4L is the primary E3 ubiquitin ligase for activated Smad2 and Smad3 in the course of transforming growth factor β (TGF-β) signal transduction (14), By regulating the turnover rate of these two key mediators of TGF-β signaling, NEDD4L plays a pivotal role in dictating both the amplitude and duration of TGF-β signal transduction (14).

TGF-β signaling, through membrane-based receptor serine/threonine kinases, controls a wide range of cellular activities including growth, differentiation, apoptosis and homeostasis. Binding of the ligand to the TGF-β receptors (TβR) induces receptor kinase-catalyzed Smad2 and Smad3 phosphorylation, creating a docking site for the co-factor Smad4 (15), which then translocates as a complex from the cytosol to the nucleus, where it recruits DNA-binding proteins to target responsive genes, setting up the canonical TGF-β pathway (16). It is generally held that TGF-β signaling has dual functionality in cancer cells. Depending on cellular context, it either suppresses or promotes tumor growth (17). Normally, epithelial cells are sensitive to growth inhibition by TGF-β and the anti-proliferative effect of TGF-β is widely assumed to be critical for its tumor-suppressor activity (18). However, malignant ovarian epithelium is resistant to the anti-proliferative effects of TGF-β (19,20). Given the existence of TGF-β in tumor microenvironment (17), the insensitivity of ovarian cancer cells to TGF-β stimulation could be one of the reasons responsible for ovarian tumor progression.

Here, we report that DDB2-mediated NEDD4L downregulation endowed a significant impact on ovarian cancer cell proliferation through TGF-β signal transduction. Our findings suggest a broader spectrum for the functions of DDB2. In particular, the connection of DDB2 to the response to signals transduced from extracellular environment provides a new focal point for exploring the contribution of DDB2 to the inhibition of ovarian cancer progression.

## MATERIALS AND METHODS

### Cell culture

Cisplatin-sensitive human ovarian cancer cell line A2780 and its derivative cisplatin-resistant subline CP70 were kindly provided by Dr Paul Modrich (Duke University, Durham, NC, USA). Three clones of CP70 cells stably transfected with pcDNA3.1-His-DDB2 (CP70-DDB2-1B, CP70-DDB2-3H and CP70-DDB2-4H) were established in our laboratory as described previously (11). SKOV3 and PEO1 ovarian cancer cell lines were provided by Dr Thomas C. Hamilton (Fox Chase Cancer Center, Philadelphia, PA, USA). These cell lines were cultured in RPMI 1640 medium supplemented with 10% fetal bovine serum (FBS), 100 μg/ml streptomycin and 100 units/ml penicillin. HeLa cells stably transfected with N-terminal FLAG-HA-tagged human DDB2 (HeLa-DDB2) were kindly provided by Dr Yoshihiro Nakatani (Dana-Farber Cancer Institute, Boston, MA, USA). HeLa and HeLa-DDB2 cell lines were cultured in Dulbecco's modified Eagle's medium supplemented with 10% FBS, 100 μg/ml streptomycin and 100 units/ml penicillin. Cells were grown at 37°C in humidified atmosphere of 5% CO_2_ in air.

### Plasmids, small interference RNA (siRNA) and cell transfection

The plasmid encoding N-terminal FLAG-tagged human *DDB2* has been described previously (21). The human *NEDD4L* cDNA was cleaved from pCMV-NEDD4L plasmid (transOMIC technologies, Huntsville, AL, USA) by using HindIII and NotI, and subcloned into pTCP vector (transOMIC) to construct pTCP-NEDD4L expression plasmid. For transient transfection, the plasmids were delivered into CP70 cells using the Lipofectamine 2000 transfection reagent according to the manufacturer's instructions (Life Technologies, Carlsbad, CA, USA). To establish a cell line with both DDB2 and NEDD4L overexpression, pTCP-NEDD4L plasmids were transfected into CP70-DDB2-3H cells, the stable transfection clone (3H + NEDD4L) was then selected by puromycin. siRNA SMARTpools designed to target human NEDD4L or DDB2 were purchased from Dharmacon (Denver, CO, USA), DDB2 siRNA #1 (5′- CAA CUA GGC UGC AAG ACU U -3′), DDB2 siRNA #2 (5′- GAU AUC AUG CUC UGG AAU U -3′) and a scramble non-targeting control siRNA (5′- UUC UCC GAA CGU GUC ACG U -3′), were synthesized by Dharmacon. A total of 100 nM siRNA was transfected into cells using Lipofectamine 2000 transfection reagent.

### Microarray analysis

Three clones of CP70 cells stably transfected with pcDNA3.1-His-DDB2 (CP70-DDB2-1B, CP70-DDB2-3H and CP70-DDB2-4H) and two clones of CP70 cells transfected with empty vectors were used for microarray analysis. Total RNA were extracted from CP70 and CP70-DDB2 cells using Trizol reagent (Life Technologies) and processed for Affymetrix transcriptsome assay using GeneChip Human transcriptome array 2.0 (Affymetrix, Santa Clara, CA, USA) at The Microarray Shared Resource of OSUCCC. Data analysis was conducted using Affymetrix transcriptsome console software. Briefly, background correction and normalization were performed and gene expression level was summarized over probes using the RMA method (22). A filtering method based on the percentage of samples with expression values below noise level (four out of five of the samples) was applied to filter out probe-sets with little or no expression. Linear regressions were used to compare the gene expression between the two types of cell lines. In order to improve the estimates of variability and statistical tests for differential expression, a variance shrinkage method was employed (23). The differentially expressed genes were claimed based on the *P*-values by controlling the average number of false positives among the tested genes.

### Co-immunoprecipitation (co-IP)

Cell lysates were prepared in RIPA buffer [10 mM Tris–HCl, pH 7.5, 200 mM NaCl, 4 mM EDTA, 1% Nonidet P-40, 1 mM PMSF and proteinase inhibitor (Roche Life Science, Indianapolis, IN, USA)] and clarified by high-speed centrifugation. For FLAG-tagged proteins, cell lysates from different samples containing equal amount of protein were incubated with 15 μl of Anti-FLAG M2 Affinity Gel (Sigma-Aldrich, St Louis, MO, USA) for 2 h at 4°C. For the analysis of endogenous protein–protein interaction, cell lysates from CP70 cells were incubated with the anti-EZH2 antibody (Cell Signaling, Danvers, MA, USA) or normal rabbit IgG, together with protein G magnetic beads (Cell Signaling) for 24 h at 4°C. Beads (gel) were washed four times with lysis buffer and boiled in the 2× sodium dodecyl sulphate (SDS) sample buffer. The eluted samples were subjected to immunoblotting for the detection of DDB2, EZH2 and/or SUZ12 with corresponding antibodies (Supplementary Table S1).

### Immunoblotting

Whole cell lysates were prepared by boiling cell pellets for 10 min in lysis buffer (2% SDS, 10% Glycerol, 62 mM Tris–HCl, pH 6.8 and a complete mini-protease inhibitor cocktail [Roche Life Science]). After protein quantification with Bio-Rad Dc Protein Assay (Bio-Rad Laboratories, Hercules, CA, USA), equal amount of proteins was loaded, separated on a polyacrylamide gel, and transferred to a nitrocellulose membrane. Protein bands were immuno-detected with appropriate antibodies, which are listed in Supplementary Table S1.

### Chromatin immunoprecipitation (ChIP) assay

The chromatin immunoprecipitation (ChIP) assay was carried out using CHIP-IT^®^ Express Enzymatic Kit (Active Motif, Carlsbad, CA, USA) with a few modifications. Immunoprecipitation (IP) was performed with various ChIP grade antibodies (Supplementary Table S1). For IP of FLAG-tagged DDB2 from HeLa-DDB2 and CP70-DDB2 cells, EZview™ Red ANTI-FLAG^®^ M2 Affinity gel (Sigma-Aldrich) was used. Immunoprecipitated DNA was purified by Phenol/chloroform extraction and quantified by real-time polymerase chain reaction (PCR) analysis with primer sets corresponding to specific regions of the NEDD4L gene promoter (Supplementary Table S2).

### Quantitative real-time reverse transcription PCR (qRT-PCR)

Total RNA was extracted using Trizol reagent (Life Technologies), and the first strand cDNA was generated by the Reverse Transcription System (Promega, Madison, WI, USA) in a 20-μl reaction containing 1 μg of total RNA. A 0.5 μl aliquot of cDNA was amplified by Fast SYBR Green PCR Master Mix (Applied Biosystems, Carlsbad, CA, USA) in each 20 μl reaction. PCR reactions were run on the ABI 7900 Fast Real-Time PCR system (Applied Biosystems) in the OSUCCC Nucleic Acid Core Facility. The primers used for the real-time RT-PCR are listed in Supplementary Table S2.

### Cell proliferation assay

For bromodeoxyuridine (BrdU) incorporation assay, CP70 and CP70-DDB2-3H cells were cultured on glass coverslips in RPMI 1640 medium supplemented with 1% FBS, treated with TGF-β (5 ng/ml) for 24 h and further cultured in the medium containing 5 μM BrdU (BD Bioscience, San Jose, CA, USA) for 4 h. The cells were fixed and permeabilized with 2% paraformaldehyde and 0.5% Triton X-100 and then denatured by incubating coverslips in 2M HCl for 10 min at 37°C. Incorporated BrdU moieties were detected by 1-h incubation with mouse-anti-BrdU (BD Bioscience, 1:200) antibody, followed by 45-min incubation with Alex Fluor 594-conjugated goat anti-mouse IgG (Life Technologies). Images were captured by using a Nikon Fluorescence Microscope E80i (Nikon, Tokyo, Japan). At least 100 cells were randomly selected for counting BrdU-positive cells.

For methylene blue assay, cells were seeded in in 96-well plates at an initial density of 500 cells/well in RPMI 1640 medium supplemented with 1% FBS. TGF-β (5 ng/ml) was added every 48 h. After 72 and 96 h, the cells were washed with phosphate buffered saline, fixed with 3.7% formaldehyde for 30 min and stained with 1.0% methylene blue for 30 min. The plate was rinsed in running water and then left to dry. One hundred microliters of solvent (10% acetic acid, 50% methanol and 40% H_2_O) was added to each well to dissolve the cells and optical density of the released color was read at 630 nm.

### Statistical analysis

Two-sample *t*-tests were used for the studies. Holm's procedure was used to control for multiple group comparisons when it is needed. *P*-values < 0.05 were considered as significant for single tests or after adjustment for multiple comparisons.

## RESULTS

### NEDD4L is targeted for downregulation by DDB2 in human ovarian cancer cells

**Figure 1.**
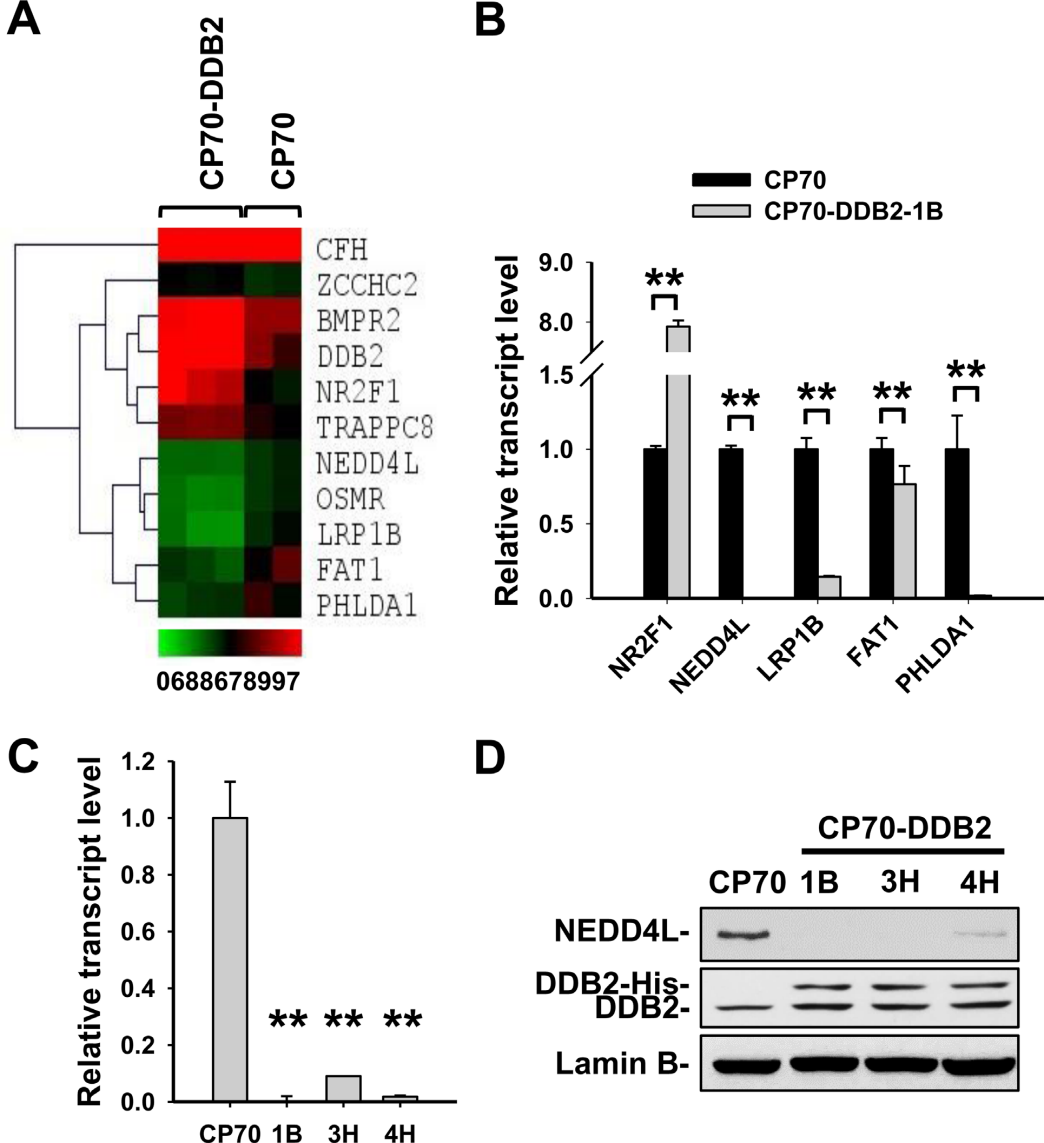
Identification of molecular targets regulated by DDB2 in the ovarian cancer cell line. (**A**) Gene expression heat map from microarray analysis of DDB2-overexprssing ovarian cancer cell lines. Total RNA was isolated from three clones of DDB2-stably expressing CP70 cells and two clones of vector-transfected CP70 cells. Microarray analysis was carried out to identify genes regulated by DDB2 overexpression. (**B**) Validation of microarray expression profiles by qRT-PCR. Real-time qRT-PCR was conducted to analyze the expression of the indicated genes in one clone of DDB2-overexpressing CP70 cells and one clone of vector-transfected CP70 cells. (**C** and **D**) Confirmation of downregulation of NEDD4L in DDB2-stably overexpressing cells. qRT-PCR (C) and immunoblotting (D) were conducted to analyze the expression of NEDD4L in three clones of DDB2-overexpressing CP70 cells and one clone of vector-transfected CP70 cells. *N* = 3, Error bars represent standard deviations (SD). **P < 0.01 compared with vector-transfected CP70 cells.

We have previously reported that the acquisition of resistance to cisplatin in ovarian cancer cell line CP70 occurs concomitantly with the loss of DDB2 expression (9,11). In addition, lower DDB2 expression is also associated with a poor outcome in patients with ovarian cancer (4). In order to understand the contribution of DDB2 as a tumor suppressor to improved prognosis in human ovarian cancer, we asked what important genes are specifically targeted by DDB2 to affect tumor progression. To address this, we performed the Affymetrix transcriptome assay with a paired group of ovarian cancer cell lines: one consisting of empty vector-transfected ovarian cancer cell line CP70 and other composed of CP70 derived CP70-DDB2 cell lines, which stably overexpress DDB2. A total of 57 genes (28 upregulated and 29 downregulated) were found to be significantly altered with DDB2 overexpression (the filtering parameters are: fold change > 2, *P*-value < 0.05) (Supplementary Tables S3 and S4). The heatmap was generated to show 11 genes that are most significantly altered (Figure 1A). The data has been deposited in the Gene Expression Ominibus (GEO, GSE66636) (www.ncbi.nlm.nih.gov/geo/).

**Figure 2.**
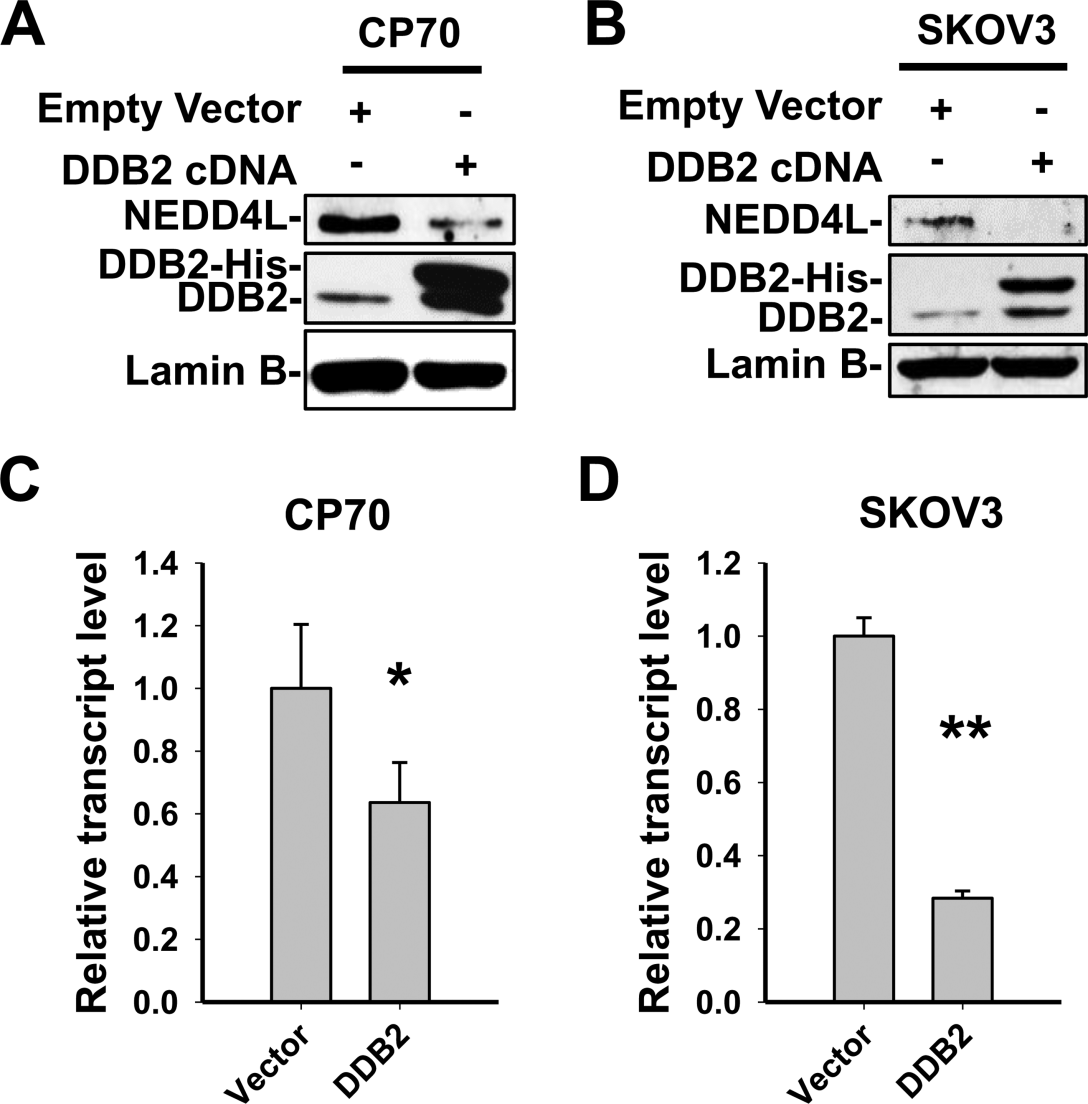
NEDD4L is downregulated by DDB2. (**A** and **B**) Immunoblotting was conducted to determine the protein level of NEDD4L in DDB2-transiently overexpressing CP70 cells (A) and SKOV3 cells (B). (**C** and **D**) qRT-PCR was conducted to analyze the mRNA level of *NEDD4L* in DDB2-transiently overexpressing CP70 cells (C) and SKOV3 cells (D). *N* = 3, Error bars represent SD. *P < 0.05; **P < 0.01 compared with vector-transfected cells.

We then selected five genes based on their importance in cell growth, proliferation and apoptosis for further validation by qRT-PCR analysis using CP70 and a representative clone of CP70-DDB2 cells (CP70-DDB2-1B). As shown in Figure 1B, the expression profile changes of all tested genes are consistent with the microarray analysis. Out of the validated genes, we are specifically interested in HECT-domain type E3 ligase *NEDD4L*, since the altered expression of this gene has been shown to affect cancer progression in a wide range of cancer types including breast cancer, glioma, non-small cell lung cancer and prostate cancer (24–31). The NEDD4L expression was further analyzed in all three DDB2 stably overexpressing CP70 cell clones (1B, 3H and 4H) at transcript and protein levels. Both qRT-PCR and immunoblotting analyses showed that NEDD4L was dramatically downregulated in all CP70-DDB2 cell lines as compared to CP70 parental cells (Figure 1C and D). To further confirm the negative regulation of NEDD4L expression by DDB2, we transiently transfected DDB2-expressing constructs into CP70 and SKOV3 ovarian cancer cell lines and showed that NEDD4L was downregulated at both protein and mRNA levels (Figure 2A–D). Similarly, transient knockdown of DDB2 in CP70-DDB2-3H and PEO1 cells using small interfering RNA (siRNA) resulted in an enhanced expression of *NEDD4L* (Supplementary Figure S1A and B). Taken together, these data indicate that *NEDD4L* is a target gene of DDB2 and DDB2 is capable of downregulating NEDD4L in ovarian cancer cells.

According to the data in Figure 2, it seems that downregulation of transcription by DDB2 overexpression is less than that of protein levels. Given that DDB2 functions as a subunit of the DDB-Cul4A E3 ubiquitin ligase (32), it is possible that DDB2 may also promote the NEDD4L protein degradation. However, knockdown of either DDB1 or Cul4A, two critical elements of the DDB-Cul4A complex, did not affect the protein level of NEDD4L (Supplementary Figure S2A), while downregulation of DDB2 dramatically increased the NEDD4L protein level (Supplementary Figure S2B). Therefore, it appears that DDB2 does not downregulate the NEDD4L protein level through the DDB-Cul4A E3 ubiquitin ligase.

### DDB2 binds to the *NEDD4L* promoter

**Figure 3.**
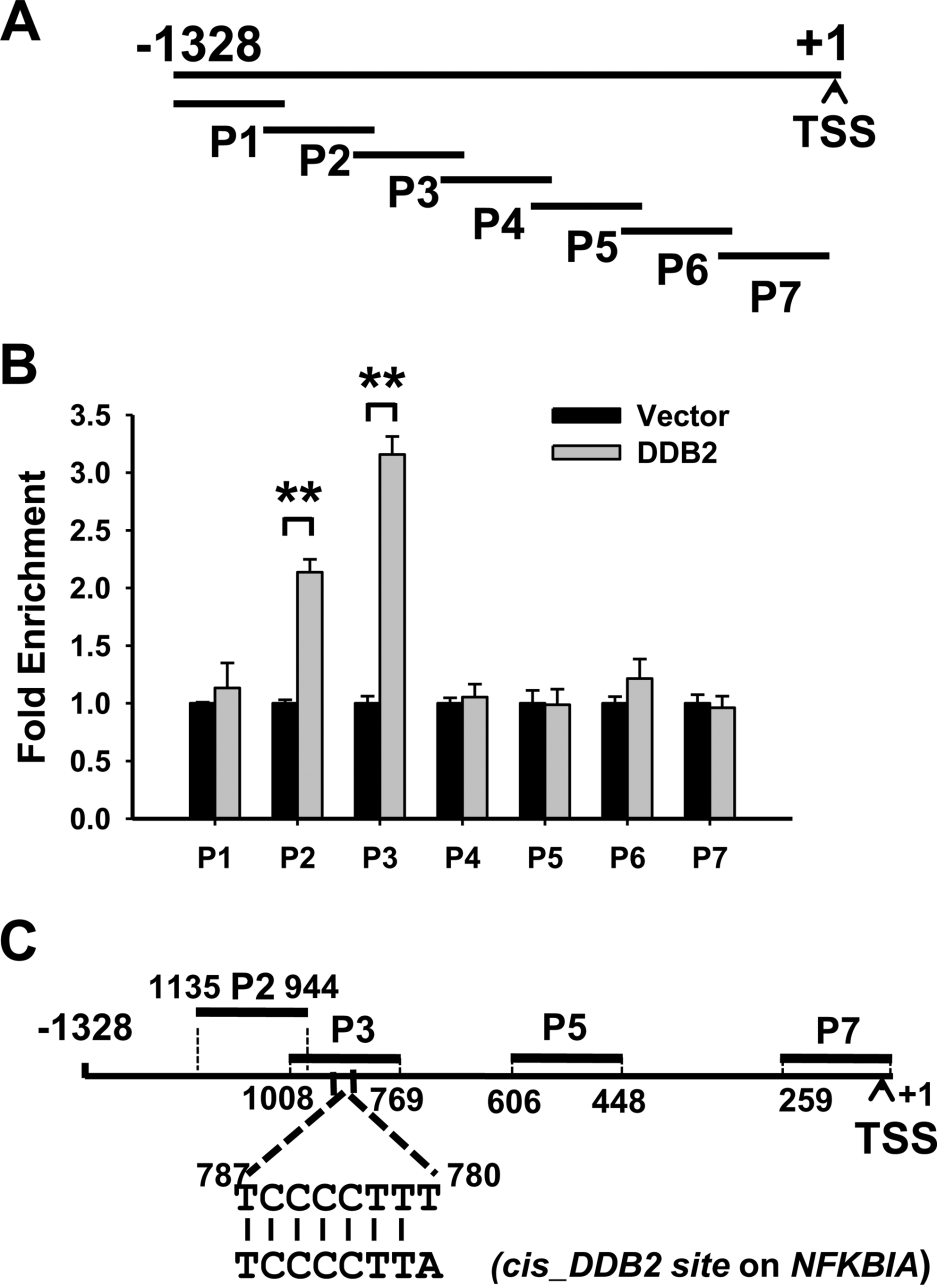
DDB2 binds to the promoter region of the *NEDD4L* gene. (**A**) The schematic representation of the human *NEDD4L* promoter region. The thick truncated lines mark the regions covered by primer sets of interest. TSS: Translation start site. (**B**) The ChIP assay was conducted to analyze the local enrichment of FLAG-tagged DDB2 across the *NEDD4L* promoter region in CP70 and FLAG-tagged DDB2-transiently transfected CP70 cells. The relative fold enrichment was quantified by normalization to input first, then normalized to CP70 cells, which is set at 1. (**C**) The schematic representation of the *NEDD4L* promoter region with a putative DDB2 binding site and the sequence alignment between the putative DDB2 binding site in the *NEDD4L* gene and that in the *NFKBIA* gene. *N* = 3, Error bars represent SD. **P < 0.01 compared with vector-transfected cells.

Accumulated evidence has shown that DDB2 functions as a transcription factor by binding to a specific *cis*-acting element in the promoter region of target genes and affects the local chromatin structure by recruiting specific transcriptional co-repressors (5,6,8–10). We hypothesized that DDB2 might employ a similar mechanism in the case of *NEDD4L* transcription control. Given that DDB2 recognizes different sites in the known target genes, we set out to examine the enrichment profile of DDB2 across the entire *NEDD4L* promoter region by ChIP analysis. FLAG-tagged DDB2 expression construct or empty vector was transiently transfected into CP70 cells and the anti-FLAG M2 affinity gel was employed to pull down DDB2 and its associated DNA fragments. To cover the whole region of the *NEDD4L* promoter, we designed seven pairs of overlapping primer sets and the promoter regions to be amplified were indicated in Figure 3A. As shown in Figure 3B, the highest enrichment of FLAG-DDB2 was identified at the P3 region of the *NEDD4L* promoter, followed by the P2 region, while no significant enrichment was found in other regions. Similar results were also obtained by using FLAG-DDB2 stably overexpressing HeLa cells, except that FLAG-DDB2 enrichment was also found in the P6 region (Supplementary Figure S3). A closer examination of the P3 region reveals a *cis*-acting element 5′-TCCCCTTT-3′ (Figure 3C), which only differs by one nucleotide from the reported DDB2 binding site (5′-TCCCCTTA-3′) in *NFKBIA* (encoding IĸBα) (5), suggesting that this site might be shared by these two genes for DDB2-mediated transcriptional regulation.

### DDB2 promotes histone H3 lysine 27 trimethylation across the *NEDD4L* promoter by recruiting EZH2

**Figure 4.**
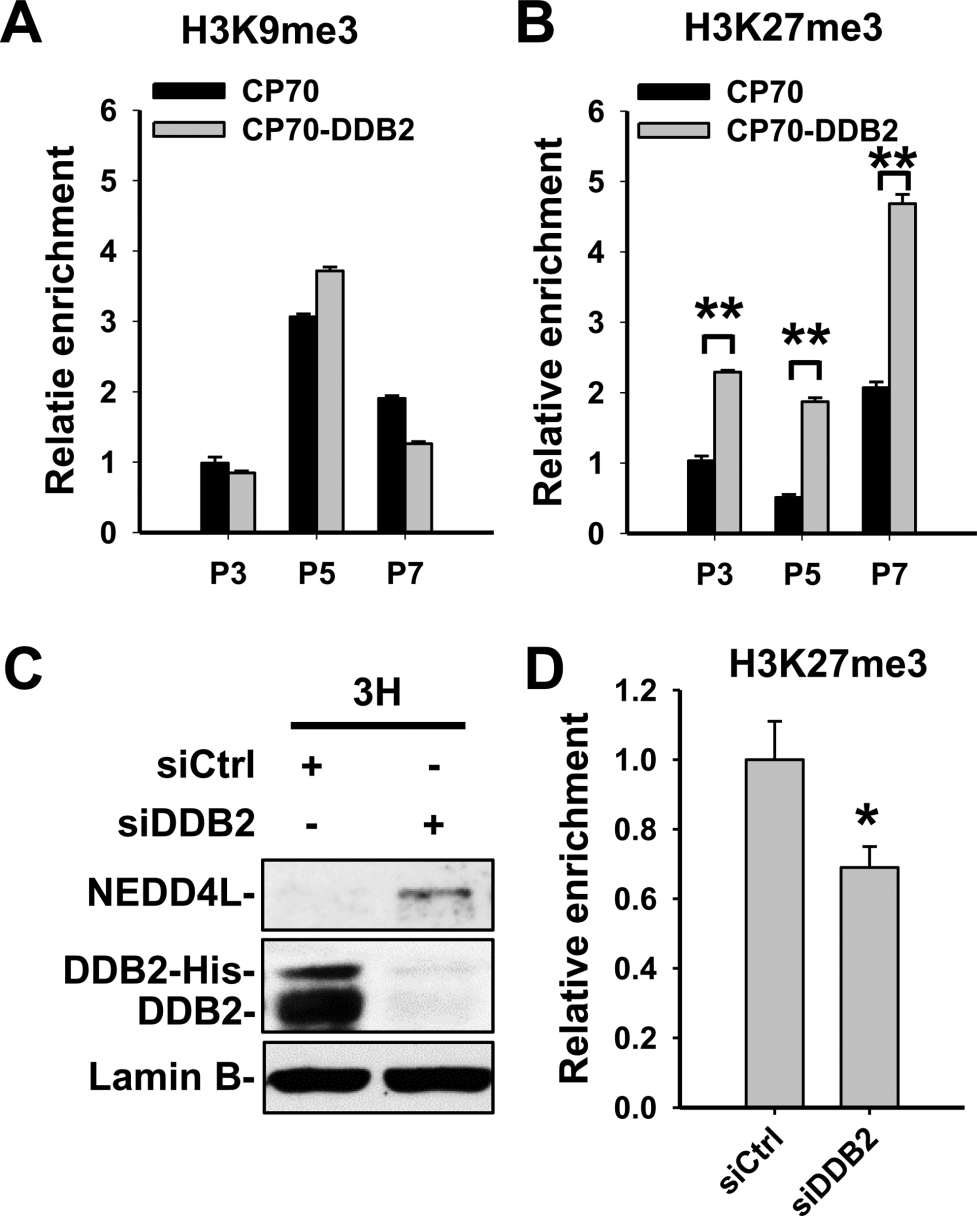
DDB2 promotes histone H3K27 trimethylation in the promoter region of the *NEDD4L* gene. (**A** and **B**) The ChIP assay was conducted to analyze the local enrichment of histone H3K9me3 (A) and H3K27me3 (B) in specific regions of the *NEDD4L* promoter, as indicated in Figure 3C, in CP70 and DDB2-overexpressing CP70 cells. The relative enrichment was quantified by normalization to input first, then normalized to the P3 of CP70 cells, which is set at 1. (**C** and **D**) DDB2 was further knocked down in DDB2-overexpressing CP70-3H cells (C), and the enrichment of H3K27me3 in the P3 region of *NEDD4L* promoter was analyzed using ChIP (D). *N* = 3, Error bars represent SD. *P < 0.05; **P < 0.01 compared with mock-transfected cells.

It has been reported that DDB2 affects histone H3 trimethylation status of multiple EMT-related genes in colon cancer (6). Thus, we focused on the analysis of the effect of DDB2 on histone trimethylation status in three selected regions of the *NEDD4L* promoter as depicted in Figure 3C. The ChIP analyses showed that DDB2 overexpression results in a significantly increased enrichment of histone H3 lysine 27 trimethylation (H3K27me3), but not H3 lysine 9 trimethylation (H3K9me3), in all three regions (Figure 4A and B). Consistently, knockdown of DDB2 using siRNA reduced the aforementioned H3K27me3 enrichment in the P3 region (Figure 4C and D). Taken together, these data suggest that local enrichment of DDB2 coincides with chromatin condensation at the potential DDB2 binding site of the *NEDD4L* promoter region, providing a previously unknown new mechanism for the DDB2-mediated *NEDD4L* transcription inhibition observed in human ovarian cancer cells.

**Figure 5.**
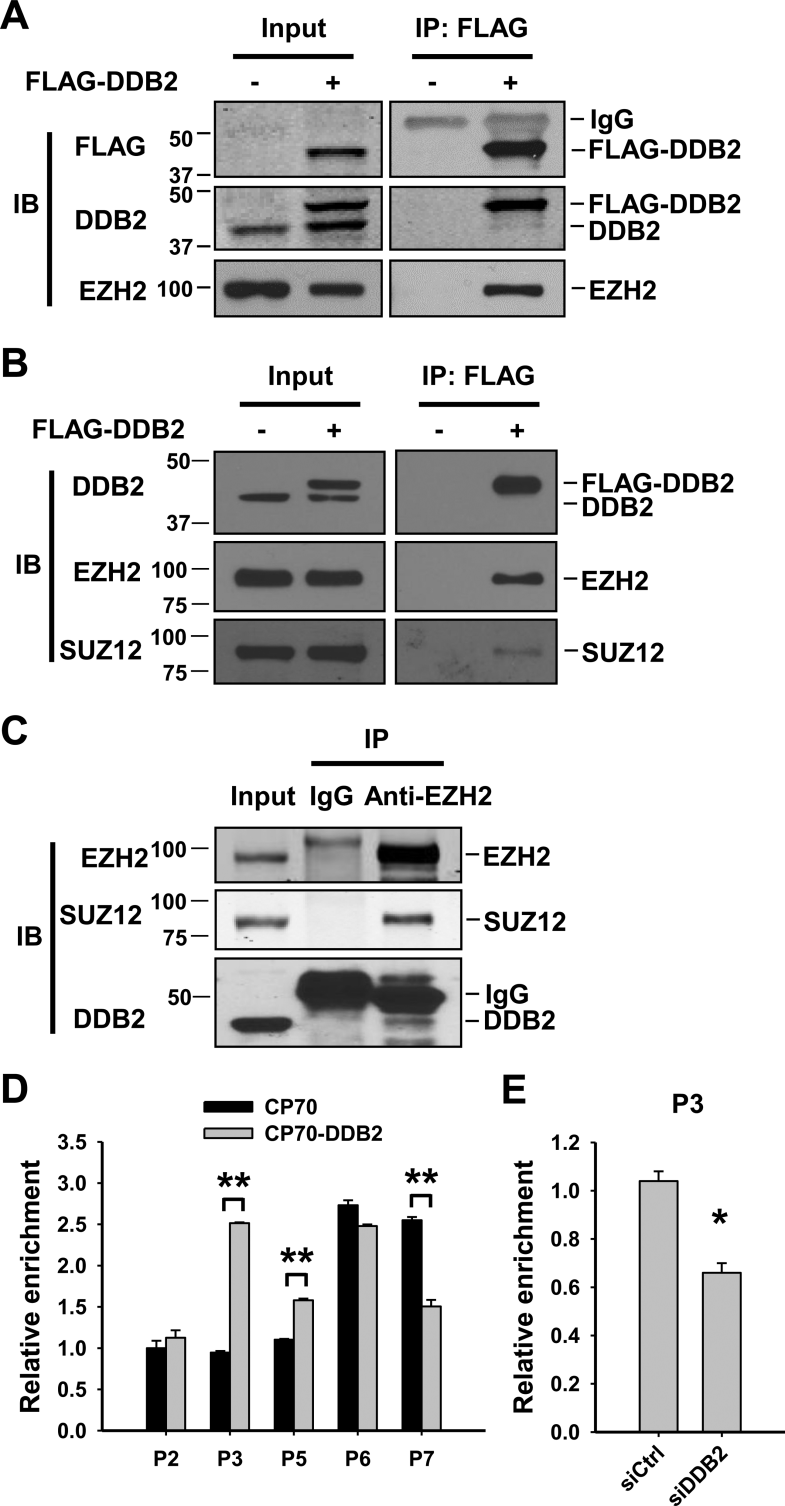
DDB2 recruits EZH2 to the *NEDD4L* promoter region. (**A** and **B**) Co-IP was carried out to show the interaction between DDB2 and EZH2 in FLAG-tagged DDB2-stably overexpressing HeLa cells (A) and FLAG-tagged DDB2-transiently overexpressing CP70 cells (B). (**C**) Co-IP was carried out in CP70 cells with the anti-EZH2 antibody to show the interaction between endogenous DDB2 and EZH2. (**D**) The ChIP assay was conducted to analyze the effect of DDB2 on the enrichment of EZH2 in the *NEDD4L* promoter region in CP70 and DDB2-overexpressing CP70-3H cells. The relative enrichment was quantified by normalization to input first, then normalized to P2 of CP70 cells, which is set at 1. (**E**) PEO1 cells were transfected with either DDB2 or Control siRNA for 48 h; the ChIP assay was conducted to analyze the enrichment of EZH2 in the P3 region of *NEDD4L* promoter. The relative enrichment was calculated as described above. *N* = 3, Error bars represent SD. *P < 0.05; **P < 0.01 compared with vector-transfected cells.

Enhancer of zeste homolog 2 (EZH2) is a histone methyltransferase that specifically catalyzes histone H3K27 trimethylation to mediate gene silencing (33,34). EZH2 interacts with embryonic ectoderm development (EED) through the WD40-containng domain of EED protein (35,36). Given that the DDB2 protein contains a 7-bladed WD40 domain (32), we reasoned that DDB2 might interact with EZH2 to recruit it to the *NEDD4L* promoter. To this end, a co-IP analysis was carried out in HeLa cells with stable FLAG-tagged DDB2 overexpression and CP70 cells with transient transfection of FLAG-tagged DDB2 by using anti-FLAG M2 affinity gel. As shown in Figure 5A and B, EZH2 could be precipitated by the anti-FLAG antibody in both HeLa and CP70 cells containing FLAG-tagged DDB2, indicating that DDB2 is able to interact with EZH2. We also confirmed the endogenous interaction by using the anti-EZH2 antibody in CP70 cells (Figure 5C). In addition, another subunit of the Polycomb Repressive Complex 2 (PRC2), SUZ12, was detected in immunocomplexes pulled down by both anti-FLAG and anti-EZH2 (Figure 5B and C), suggesting that DDB2 is able to interact with the PRC2.

We then determined whether EZH2 can be recruited to the *NEDD4L* promoter by DDB2. ChIP analysis demonstrated a significantly increased enrichment of EZH2 in P3 and P5 regions of the *NEDD4L* promoter in DDB2-overexpressing CP70 cells compared to their parental CP70 cells (Figure 5D), while knockdown of DDB2 reduced the enrichment of EZH2 in the P3 region in PEO1 cells (Figure 5E). These data indicate that DDB2 facilitates the enrichment of EZH2 at the *NEDD4L* promoter by protein–protein interaction. However, we also noticed a significantly decreased enrichment of EZH2 in the P7 region in DDB2-overexpressing CP70 cells. Given that DDB2 was not found to bind to the P7 region (Figure 3B), the reduced EZH2 enrichment may not be attributed to the direct effect of DDB2 overexpression.

### DDB2 enhances the TGF-β signaling through downregulation of NEDD4L

**Figure 6.**
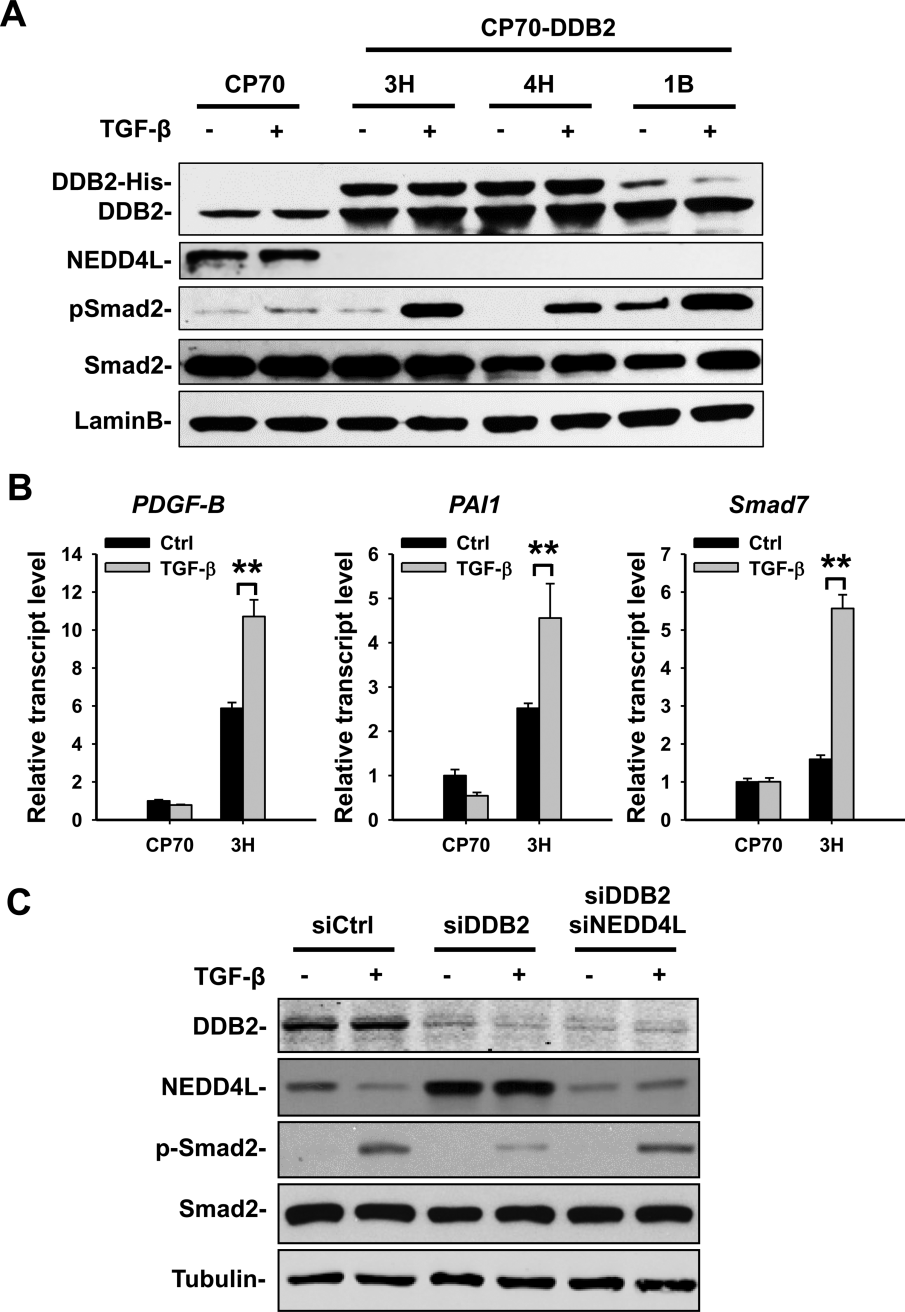
DDB2 modulates the TGF-β signaling *via* NEDD4L in human ovarian cancer cells. (**A**) Immunoblotting analysis shows that TGF-β-induced Smad2 phosphorylation is enhanced in DDB2-overexpressing CP70 cells. CP70 and three clones of DDB2-stably overexpressing CP70 cells were treated with TGF-β (5 ng/ml) for 4 h. Total cell lysates were prepared and subjected to immunoblotting analysis to determine the phosphorylation of Smad2. (**B**) qRT-PCR was conducted to analyze the TGF-β target genes. CP70 and CP70-DDB2 cells were treated with TGF-β for 4 h and total RNA was isolated for qRT-PCR analysis. (**C**) Concomitant knockdown of DDB2 and NEDD4L rescued the DDB2 downregulation-induced reduction of TGF-β signaling. DDB2 was knocked down alone or together with NEDD4L using their corresponding siRNA in PEO1 cells and treated with TGF-β for 4 h. Immunoblotting was carried out to detect the phosphorylation of Smad2. *N* = 3, Error bars represent SD. **P < 0.01 compared with non-TGF-β treated cells.

As a HECT-type E3 ligase (37), NEDD4L has been shown to control the duration and intensity of TGF-β signaling by targeting phosphorylated Smad2, the key mediator of activated TGF-β signaling cascade, to proteasomal degradation (14). Having established that DDB2 is responsible for NEDD4L transcription repression in human ovarian cancer cells, we asked whether this might be translated into alteration in TGF-β signaling and responses of target genes. To this end, we treated CP70 cells and three clones of CP70-DDB2 cells with TGF-β and examined (i) the expression level of phosphorylated Smad2, the hallmark of TGF-β activation, by immunoblotting and (ii) the transcript levels of representative TGF-β target genes by qRT-PCR analysis. As shown in Figure 6A, CP70 cells which are low in DDB2 but high in NEDD4L expression, exhibited undetectable change in phosphorylated Smad2 following TGF-β stimulation. However, in the presence of DDB2 overexpression, cells displayed a marked induction of phosphorylated Smad2 in response to the same treatment, indicating that DDB2 sensitizes cells to a more active reaction to TGF-β stimulation. In this context, DDB2-mediated NEDD4L downregulation is associated with a robust induction of three representative TGF-β target genes, e.g. platelet-derived growth factor beta (*PDGF-B*), plasminogen-activator inhibitor 1 (*PAI1*) and *Smad7* (Figure 6B). Next, we used another human ovarian cancer cell line PEO1 to further examine the coordination between DDB2 and NEDD4L in the regulation of TGF-β signaling transduction. As shown in Figure 6C, the NEDD4L protein level increased concomitantly with DDB2 knockdown by siRNA, indicating that the DDB2-mediated transcriptional repression of NEDD4L is lifted. Consequently, TGF-β mediated Smad2 phosphorylation in these DDB2-knockdown cells was compromised. When both NEDD4L and DDB2 were simultaneously knocked down, the attenuated TGF-β-induced phosphorylation of Smad2 restored to the control level (Figure 6C). Taking together, these data indicate that DDB2-NEDD4L-pSmad2 axis acts in human ovarian cancer cells to modulate the TGF-β signal propagation.

### DDB2 overexpression enhanced the anti-proliferative effect of TGF-β stimulation through downregulation of NEDD4L

**Figure 7.**
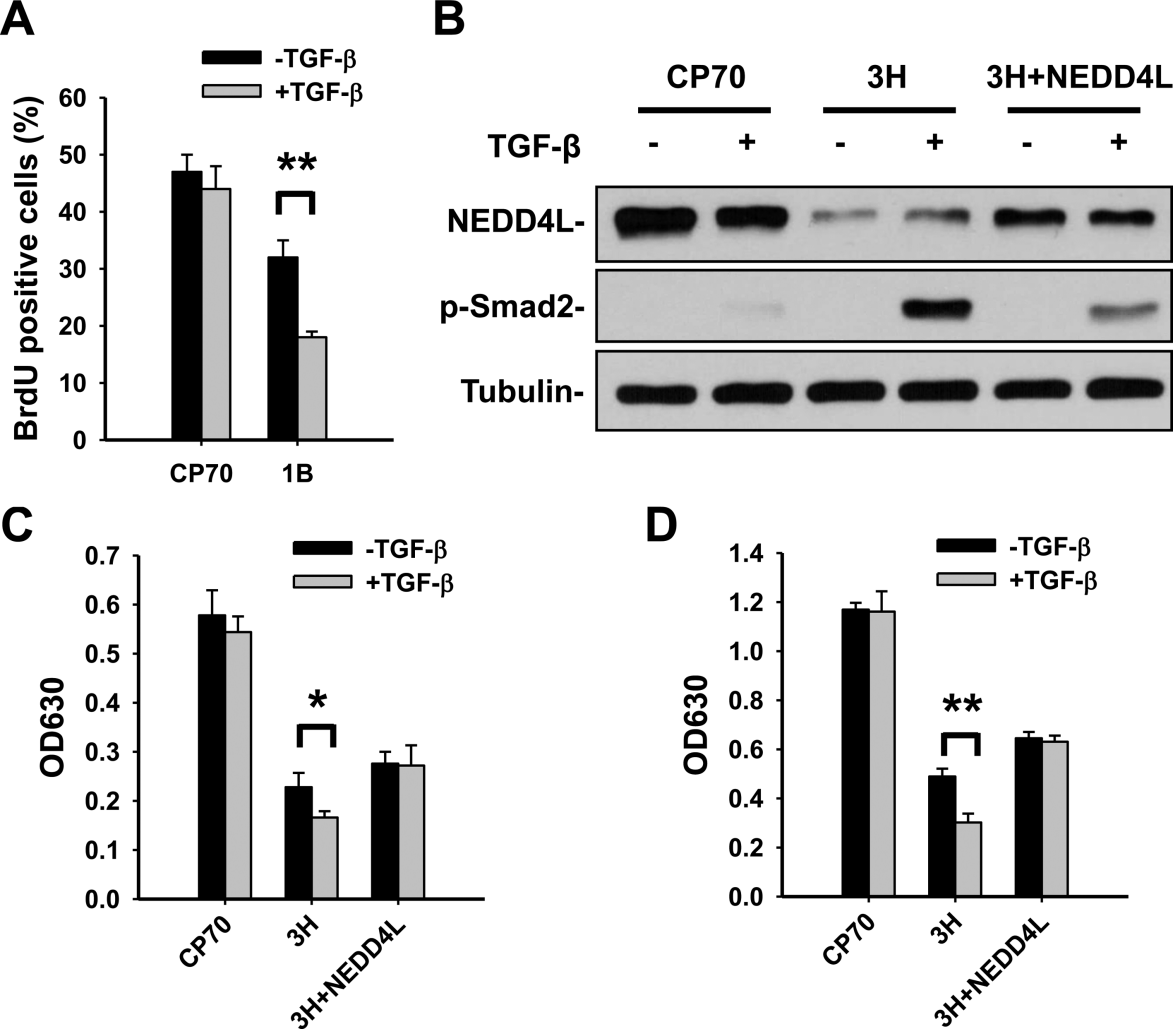
DDB2 promotes TGF-β-induced ovarian cancer cell growth inhibition through downregulation of NEDD4L. (**A**) Overexpression of DDB2 sensitized CP70 cells to TGF-β-induced cell proliferation inhibition. CP70 and DDB2-stably overexpressing CP70-3H cells were treated with TGF-β for 2 days, and the BrdU incorporation assay was conducted to determine the cell proliferation. (**B**) Overexpression of NEDD4L in DDB2-overexpressing cells compromised TGF-β signaling in ovarian cancer cells. NEDD4L was stably overexpressed in DDB2-stably overexpressing CP70-3H cells (3H + NEDD4L) and treated with TGF-β for 4 h. Immunoblotting analysis was conducted to determine the phosphorylation of Smad2. (**C** and **D**) CP70, CP70-DDB2-3H (3H) and CP70-DDB2 cells with NEDD4L stable overexpression (3H + NEDD4L) were treated with TGF-β for 72 h (C) and 96 h (D). Cell growth was measured using the methylene blue assay. *N* = 5, Error bars represent SD. *P < 0.05; **P < 0.01 compared with non-TGF-β treated cells.

Ovarian cancer cells are known to lose responsiveness to inhibitory growth signals exerted by TGF-β (19,20). Given that most ovarian cancers have reduced expression of DDB2 (4) and our current finding that DDB2 enhances TGF-β signaling transduction in ovarian cancer cell lines, we sought to understand whether DDB2 enhances TGF-β-induced ovarian cancer cell growth inhibition. We compared the behavior of DNA synthesis in CP70 and DDB2-overexpressing CP70 cells (3H) following TGF-β stimulation by the BrdU incorporation assay. As shown in Figure 7A, no apparent change in BrdU incorporation was observed in CP70 cells before and after TGF-β addition, which further confirmed the low sensitivity of this cell line to TGF-β induced effects. However, TGF-β treatment significantly reduced the BrdU-positive cell fraction in DDB2-overexpressing CP70 cells, indicating that DDB2 is able to sensitize ovarian cancer cells to the growth inhibitory effect of TGF-β. To further understand whether this effect is mediated through NEDD4L, we stably overexpressed NEDD4L in DDB2-overexpressing CP70 cells (3H + NEDD4L) and determined cell proliferation rate in response to TGF-β. As shown in Figure 7B, overexpression of NEDD4L compromised TGF-β-induced phosphorylation of Smad2 in DDB2-overexpressing CP70 cells. When these cells were treated with TGF-β for either 72 or 96 h, DDB2-overexpressing CP70 cells (3H) displayed slower proliferation compared to non-TGF-β treated cells. In contrast, further overexpression of NEDD4L in 3H cells (3H + NEDD4L) diminished the sensitivity of 3H cells to TGF-β-induced cell proliferation inhibition (Figure 7C and D). These data support our primary hypothesis that DDB2 sensitize ovarian cancer cells to TGF-β-induced cell growth inhibition though downregulation of NEDD4L.

## DISCUSSION

Increasing evidence has emerged to support the function of DDB2 in transcriptional regulation. Besides the reported genes, such as *MnSOD*, *catalase*, *IκBα*, *VEGF*, *Zeb1*, *Snail* and *Bcl-2*, additional genes were identified in our microarray analysis to be targeted and regulated by DDB2, e.g. *NEDD4L*. We also showed that DDB2 enhances the TGF-β signaling through downregulation of NEDD4L expression in ovarian cancer cells, providing a novel mechanism for the loss of response to TGF-β-induced growth inhibition in ovarian cancer cells (19,20) that always exhibit low DDB2 expression level (4).

DDB2 has been demonstrated to bind to the promoter region of its target genes. Nevertheless, depending upon the context and downstream function, DDB2 employs different mechanisms to regulate gene transcription. For instance, DDB2 recruits Suv39h histone methyltransferase onto the promoters of *MnSOD*, *catalase*, *VEGF*, *Zeb1* and *Snail* to repress their expression by increasing histone H3K9 trimethylation (6,10). Whereas, DDB2 brings histone deacetylase 1 (HDAC1) to the *Bcl-2* promoter to repress Bcl-2 expression by deacetylating histones H3K9 and H3K14 (9). However, no differences were observed in the acetylation and methylation of H3K27 and H3K4 in the promoter region of *IκBα* gene in MDA-MB231 cells expressing or not expressing DDB2 (5). In an attempt to explain the role of DDB2 in the suppression of the *NEDD4L* gene, we examined whether the DDB2-dependent decrease in NEDD4L expression is regulated at the epigenetic level. Interestingly, our data suggests a uniquely different mechanism for DDB2-mediated repression of gene expression, i.e. DDB2 recruits EZH2 histone methyltransferase to the *NEDD4L* promoter region to increase the histone H3K27 trimethylation, which is instrumental in the repression of *NEDD4L* transcription.

DDB2 seems to be preferably enriched in an 8-bp long *cis*-acting element located 780 bp upstream of the *NEDD4L* transcription start site. This site differs in only one nucleotide from the reported DDB2-binding site in the *IκBα* promoter. However, DDB2 exerts opposite effects on the transcription of *NEDD4L* and *IκBα*. Although DDB2 represses both *NEDD4L* and *MnSOD* transcription, the binding sites of DDB2 in the gene promoters of these two genes are different, reflecting the versatility of DDB2 in transcriptional control.

*NEDD4L* has emerged as an interesting and important target gene of DDB2 as it is found to be associated with the prognosis of ovarian cancer patients. The analysis of public database of gene expression arrays (http://kmplot.com/analysis/) reveals that ovarian cancer patients with higher expression of *NEDD4L* suffered a lower progression-free survival for stage 3–4 patients than those with lower *NEDD4L* expression (Supplementary Figure S4), indicating an association between high *NEDD4L* expression and poor outcome for ovarian cancer patients at advanced stages. Given that NEDD4L is the principle ubiquitin ligase targeting activated Smad2/3 to constrain the signaling capacity of the TGF-β pathway (14), it is likely that NEDD4L impacts the progression of ovarian cancer through compromising the responsiveness of cancer cells to TGF-β stimulation.

In the canonical TGF-β pathway, TGF-β binds to TβR, results in the phosphorylation of the Type I receptor (TβR-I), which further recruits and phosphorylates Smad2 and Samd3. The phosphorylated Smad2/Smad3 form heteromeric complexes with the common partner Samd4 and translocate into the nucleus to regulate gene expression (17). The effect of TGF-β on ovarian cancer cells have been evaluated on tumor growth, metastasis and most recently on the tumor microenvironment (19,20,38,39). Ovarian cancer cells are known to have lost their responsiveness to inhibitory growth signals exerted by TGF-β (19,20). It is proposed that resistance to TGF-β-induced growth inhibition originates from downstream of TβR-induced phosphorylation, since the initial steps in the TGF-β signaling pathway, from receptor expression down to TβR-I phosphorylation, remain intact in primary ovarian cancer cells (19). Here, in this study, we have demonstrated that in DDB2-deficient ovarian cancer cells, phosphorylated Smad2 was hardly increased by TGF-β treatment. However, if we downregulated NEDD4L by upregulation of DDB2, TGF-β-induced phosphorylation of Smad2 increased significantly and the cells became responsive to TGF-β-induced cell growth inhibition. These findings suggest that resistance to TGF-β-induced growth inhibition originates, at least partially, from the overwhelming turnover of activated Smad2/Smad3 through enhanced expression of NEDD4L. Thus, by regulating NEDD4L expression, DDB2 enters as a key upstream regulator of the TGF-β signaling cascade in ovarian cancer cells.

Our findings have revealed, for the first time, that DDB2 is actively involved in regulating the response of ovarian cancer cells to TGF-β stimulation *via* NEDD4L. We also showed that one of the downstream effects of this regulation is on tumor growth. It has been recently revealed that the emergence of TGF-β gene signature is closely associated with the progression and invasiveness of ovarian cancer (38). In light of the importance of NEDD4L in the regulation of TGF-β signal transduction and the importance of TGF-β signal transduction pathway in ovarian carcinogenesis, it will be of great interest in the near future to address whether there exists a subset of DDB2-induced TGF-β signature genes in this cancer type and whether DDB2 can sensitize primary ovarian cancer cells to the stimulation of TGF-β.

## Supplementary Material

SUPPLEMENTARY DATA
