# Supplementary material for: DDB2 modulates TGF-β signal transduction in human ovarian cancer cells by downregulating NEDD4L
Source: Nucleic Acids Res. 2015 Jun 29;43(16):7838–49. doi: 10.1093/nar/gkv667 (PMC4652750; doi:10.1093/nar/gkv667)
Supplement: SUPPLEMENTARY DATA [file supp_43_16_7838__index.html]

DDB2 modulates TGF-β signal transduction in human ovarian cancer cells by downregulating NEDD4L — SUPPLEMENTARY DATA 

# DDB2 modulates TGF-β signal transduction in human ovarian cancer cells by downregulating NEDD4L

## SUPPLEMENTARY DATA

- SUPPLEMENTARY DATA
