# Supplementary material for: DDB2 modulates TGF-β signal transduction in human ovarian cancer cells by downregulating NEDD4L
Source: Nucleic Acids Res. 2015 Jun 29;43(16):7838–49. doi: 10.1093/nar/gkv667 (PMC4652750; doi:10.1093/nar/gkv667)
Supplement: SUPPLEMENTARY DATA [file supp_gkv667_nar-01271-v-2015-File003.pdf]

DDB2 modulates TGF-β signal transduction in human ovarian cancer cells by downregulating NEDD4L

Zhao, et al

Supplementary Figures

Figure S1. Transient downregulation of DDB2 enhances the transcription level of NEDD4L in ovarian cancer cell lines. Two different siRNAs sequences targeting DDB2 were transiently transfected into CP70-DDB2-3H cells (A), and siRNA SMARTpool designed to target human DDB2 were transiently transfected into PEO1 cells (**B**). After 48 h, total RNA was isolated and the mRNA levels of DDB2 and NEDD4L were detected using qRT-PCR. N = 3, Error bar: standard deviation. **: *P* < 0.01 compared with control siRNA-transfected cells.

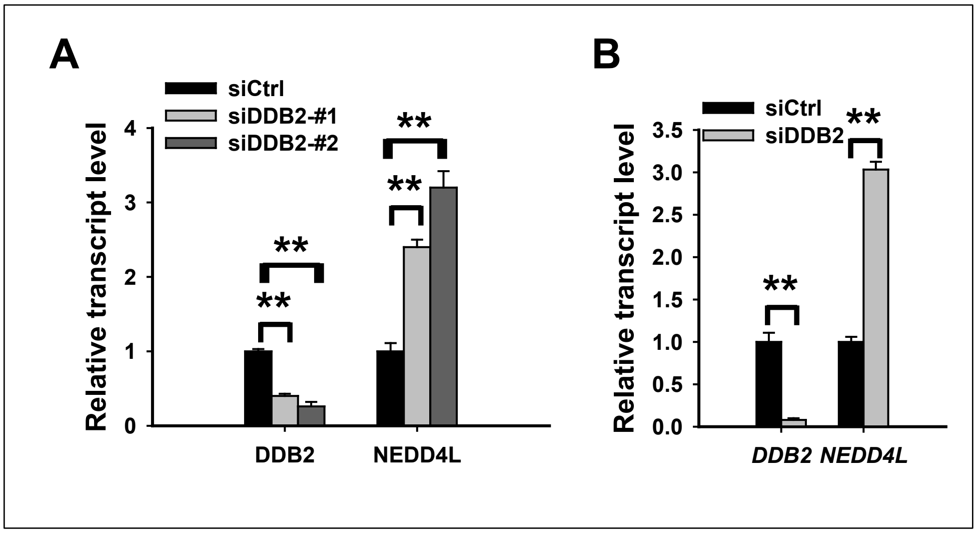

Figure S2. DDB-Cul4A E3 ubiquitin ligase is not involved in DDB2-mediated downregulation of NEDD4L. PEO1 cells were transfected with siRNA SMARTpool designed to target DDB1, Cul4A, DDB2, or siCtrl for 48 h. Whole cell lysates were prepared and subjected to immunoblotting to detect the protein level of NEDD4L. DDB1, Cul4A, and DDB2 were detected to show the knockdown efficiency. Tubulin was detected as a loading control.

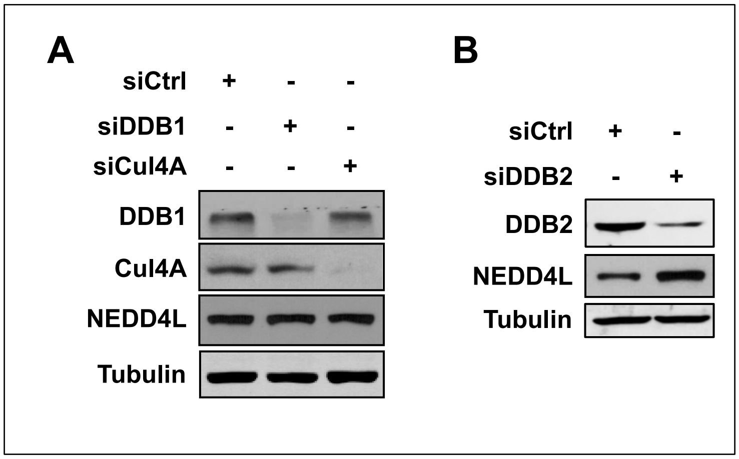

Figure S3. DDB2 binds to the promoter region of the NEDD4L gene. The ChIP assay was conducted to analyze the local enrichment of FLAG-tagged DDB2 across the *NEDD4L* promoter region in HeLa and FLAG-tagged DDB2 stably transfected HeLa cells (HeLa-DDB2). **: $P < 0.01$ compared with control siRNA-transfected cells.

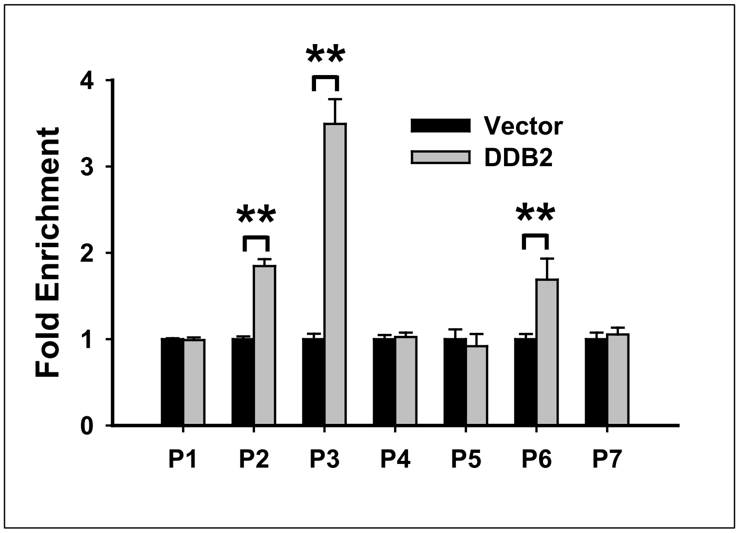

Figure S4. Prognostic significance of NEDD4L in ovarian cancer. The correlation of *NEDD4L* mRNA expression and progression-free survival (PFS) of ovarian cancer patient was analyzed using KM-plotter (http://kmplot.com/analysis/). The median expression value of *NEDD4L* was used as the cutoff, the patients were dichotomized into *NEDD4L*-high- or *NEDD4L*-low-expression groups. The effect of *NEDD4L* mRNA expression level on the PFS in 1511 ovarian cancer patients (**A**), and 1071 ovarian cancer patients at stage 3 or 4 (**B**) were analyzed and the Kaplan-Meier plots were generated.

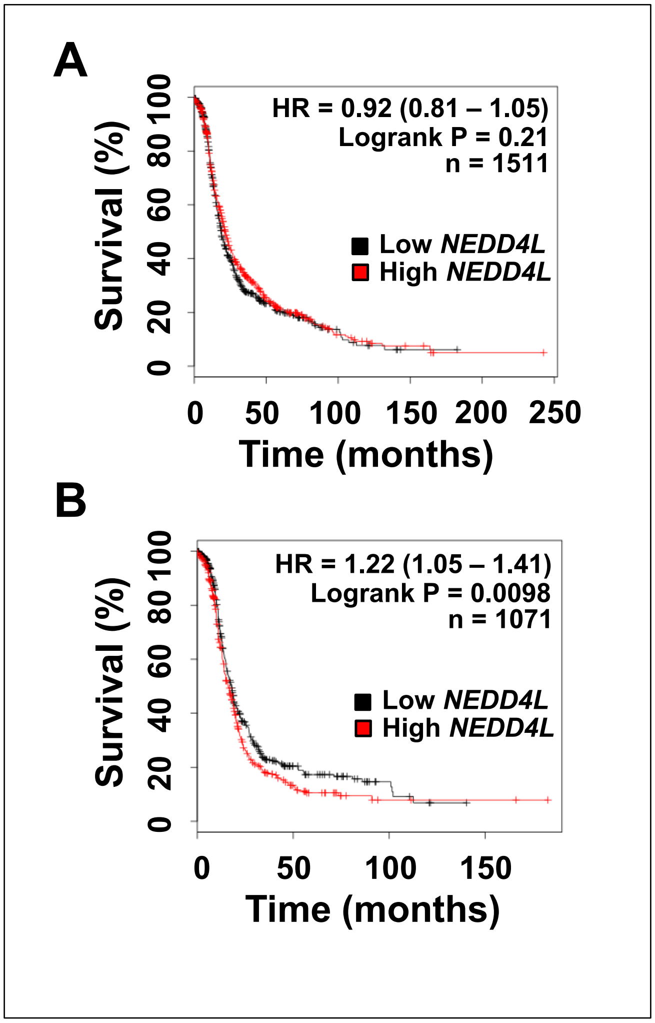

Supplementary Table S1. Antibodies used for ChIP, IP and Immunoblotting

Name	Catalog number	Company
**For ChIP and IP**		
anti- Histone H3K27me3 antibody	39155	Active Motif
anti- Histone H3K9me3 antibody	39161	Active Motif
EZview™ Red ANTI-FLAG® M2 Affinity gel	F2426	Sigma
Anti-EZH2 antibody	5246	Cell Signaling
Normal rabbit IgG	sc-2027	Santa Cruz
**For Immunoblotting**		
anti-DDB2	AF3297	R & D Systems
anti-DDB1	sc-25367	Santa Cruz
Anti-Cul4A	2699	Cell Signaling
anti-NEDD4L	Ab131167	Abcam
anti-pSmad2	3108	Cell Signaling
anti-Smad2	5339	Cell Signaling
Anti-EZH2	5246	Cell Signaling
Anti-SUZ12	3737	Cell Signaling
Anti-Lamin B	sc-6216	Santa Cruz

Supplementary Table S2. Primers for ChIP and RT-PCR

PCR primers	Forward	Reverse	Length
**RT-PCR**			
LRP1B	TTCCCAAGGCCTGGATCAAC	GCTTCCTGACAGCACCATCT	274
PHLDA1	GCAGGGCATTTGCTAAGTGG	GAAGGCTGACCTCGGAATGT	268
NR2F1	TTCTTCAAGAGGAGCGTCCG	ATGTAGCCGGACAGGTAGCA	242
FAT1	CGGGTGAGCTCCACGAGA	CCTTACTTCCCACGCTGGAT	279
NUPR1	CCCACTTCACCTCTGACTCC	TCCGCAGTCCCGTCTCTATT	235
PAI1	CCTGGTTCTGCCCAAGTTCT	ATGCGGGCTGAGACTATGAC	225
PDGF	GAGTCGGCATGAATCGCTGC	CCCATCTTCCTCTCCGGGGT	179
Smad7	TCGGAAGTCAAGAGGCTGTG	GACAGTCTGCAGTTGGTTTGAG	163
NEDD4L	AGAAACTGCCCAGAGCTCAC	TCGCCTCTGCAAAAGTCTGT	213
DDB2	CTCCTCAATGGAGGGAACAA	GTGACCACCATTCGGCTACT	132
GAPDH	AAGGTGAAGGTCGGAGT	GAAGATGGTGATGGGATTTC	226
**ChIP-PCR**			
**P1 (-1328 to -1118)**	GGCAAATGGAATGTTCTGCG	AGCCACCGTAGGATTCTTCC	211
**P2 (-1135 to -944)**	AGAGAAACGGTAGCTGGGTG	CCTTGCAGGGAACAAAACCA	192
**P3 (-1008 to -769)**	TCTGTTTTCGCGTGGGTG	TGAGCTCGCTGTTAACTGTG	240
**P4 (-812 to -592)**	AAGGGGTCTAGAAAATCC	GTACTGGCGCGCTTG	221
**P5 (-606 to -448)**	CCAAGCGCGCCAGTACAC	CCCCAAGCCAATCACAGG	159
**P6 (-511 to -244)**	CTGAACTCCGCCACCC	GCTTTCCCTCCCCGCG	268
**P7 (-259 to -1)**	TCGCGGGGAGGGAAAG	GTTCTCCCCTCTGCCTC	259

Supplementary Table S3. Genes upregulated by DDB2 in ovarian cancer cells identified by microarray analysis.

CP70-DDB2: Averaged expression values in log scale in DDB2-overexpressing CP70 cells

CP70-Vector: Averaged expression values in log scale in Vector-transfected CP70 cells

DDB2 - Vector: Differences in logged expression values between DDB2-overexpressing and Vector-transfected CP70 cells

FC(DDB2/vector): Fold changes in the gene expression in DDB2-overexpressing cells over Vector-transfected cells

P-values: Statistic significance of the gene expresson difference between two groups

Number	Gene ID	CP70-DDB2	CP70-Vector	DDB2 - Vector	FC(DDB2/vector)	P-values
1	NR2F1	8.43908	6.49272	1.94636	3.85400	0.00131
2	ZCCHC2	6.75781	5.72978	1.02803	2.03923	0.00308
3	DDB2	9.33286	7.54524	1.78763	3.45247	0.00333
4	NAP1L3	7.09084	5.48058	1.61026	3.05307	0.00487
5	TMX4	6.66634	5.65746	1.00888	2.01235	0.01291
6	LOC729987	5.43987	4.15671	1.28315	2.43370	0.01475
7	DPYD	6.89591	4.99416	1.90175	3.73667	0.01550
8	LRRK2	5.71579	4.14246	1.57333	2.97591	0.01726
9	KIAA0825	6.63242	4.22536	2.40706	5.30391	0.02019
10	IFT57	8.11430	6.33978	1.77452	3.42123	0.02151
11	FREM2	5.50494	4.32894	1.17600	2.25950	0.02159
12	HMGN5	8.36013	7.06106	1.29906	2.46069	0.02172
13	MYOF	9.55043	8.46901	1.08141	2.11611	0.02575
14	ZBTB20	5.92229	4.57138	1.35091	2.55074	0.02608
15	EPG5	6.68866	5.67341	1.01525	2.02125	0.02793
16	NID1	8.35794	6.94295	1.41499	2.66658	0.02804
17	PCDH9	7.17695	4.49485	2.68210	6.41791	0.02877
18	C16orf45	7.53985	5.52280	2.01705	4.04755	0.03009
19	AKR1E2	6.39908	5.13962	1.25945	2.39405	0.03147
20	PIK3C3	6.92813	5.67162	1.25651	2.38917	0.03153
21	SNX7	8.01868	6.29648	1.72220	3.29939	0.03265
22	LPIN1	7.73973	6.72916	1.01057	2.01470	0.03446
23	TTC18	5.83853	4.67994	1.15858	2.23238	0.03553
24	PLK2	8.66680	6.16034	2.50645	5.68221	0.03947
25	LINC00478	7.00723	4.81966	2.18757	4.55538	0.04458
26	CRYBG3	6.26126	4.75522	1.50604	2.84030	0.04491
27	FBN1	7.76125	6.28781	1.47344	2.77684	0.04672
28	PAM	7.38977	6.05614	1.33363	2.52036	0.04789

Supplementary Table S4. Genes downregulated by DDB2 in ovarian cancer cells identified by microarray analysis

CP70-DDB2: Averaged expression values in log scale in DDB2-overexpressing CP70 cells

CP70-Vector: Averaged expression values in log scale in Vector-transfected CP70 cells

Vector - DDB2: Differences in logged expression values between Vector-transfected and DDB2-overexpressing CP70 cell

FC(vector/DDB2): Fold changes in the gene expression in Vector-transfected cells over DDB2-overexpressing cells

P-values: Statistic significance of the gene expresson difference between two groups

Number	Gene ID	CP70-DDB2	CP70-Vector	Vector-DDB2	FC(vector/DDB2)	P-values
1	OSMR	3.54755	5.71205	2.16450	4.48312	0.00063
2	NEDD4L	4.07769	5.69877	1.62108	3.07604	0.00089
3	LRP1B	3.23961	6.17455	2.93494	7.64723	0.00138
4	FAT1	5.02133	7.21550	2.19417	4.57625	0.00248
5	PHLDA1	5.37162	6.94212	1.57050	2.97008	0.00383
6	LAMB1	4.53014	5.78686	1.25672	2.38952	0.00428
7	FAM102B	4.19487	6.34290	2.14803	4.43222	0.00600
8	KIRREL	5.26075	7.05788	1.79713	3.47527	0.00763
9	LGALS3BP	6.21287	7.53004	1.31717	2.49178	0.00766
10	ALDH1A1	6.51544	10.79940	4.28396	19.48050	0.00781
11	NRIP3	4.45797	5.91363	1.45566	2.74282	0.00841
12	UBASH3B	3.86962	5.93005	2.06043	4.17111	0.00940
13	VEGFC	4.16891	6.35076	2.18185	4.53736	0.01192
14	COTL1	6.41494	7.87600	1.46106	2.75311	0.01195
15	GLIPR1	4.37945	6.20285	1.82340	3.53914	0.01458
16	MT1F	4.73071	5.98237	1.25166	2.38115	0.01514
17	DHRS3	6.07603	7.15586	1.07984	2.11380	0.01522
18	SLC25A24	4.14478	5.61335	1.46857	2.76747	0.01813
19	LHX2	5.30145	6.63227	1.33082	2.51546	0.02569
20	PDZD2	5.41908	6.90152	1.48245	2.79422	0.02798
21	NT5C2	8.13125	9.35467	1.22342	2.33499	0.03063
22	SEL1L3	5.32019	6.40397	1.08378	2.11958	0.03456
23	ATP1B1	6.44373	8.12073	1.67700	3.19763	0.03528
24	DUSP6	4.25347	7.41180	3.15833	8.92799	0.03680
25	POPDC3	5.98273	7.17892	1.19619	2.29133	0.03890
26	PTGS2	3.45214	5.13519	1.68305	3.21107	0.04367
27	EPCAM	5.41782	6.55946	1.14164	2.20631	0.04439
28	GCNT4	3.95954	5.92579	1.96625	3.90752	0.04841
29	SIM2	4.43725	5.48309	1.04584	2.06457	0.04863
